# Comment on “Reversible 3D optical data storage and information encryption in photo-modulated transparent glass medium”

**DOI:** 10.1038/s41377-022-00919-0

**Published:** 2022-07-26

**Authors:** Gael Poirier, Marcelo Nalin, Sidney J. L. Ribeiro, Younes Messaddeq

**Affiliations:** 1 Institute of Science and Technology, Federal University of Alfenas, Campus Poços de Caldas, Poços de Caldas, MG Brazil; 2 Institute of Chemistry, São Paulo State University UNESP, Araraquara, SP Brazil

**Keywords:** Optical data storage, Integrated optics

Dear Editor,

The development of reversible photochromic materials combining efficient writing/erasing properties, low-cost and environmentally friendly compositions is a breakthrough challenge and a subject of many research works in the last decades. In this perspective, transparent oxide glasses are promising since their production is ensured by a simple melt-quenching technique and a proper laser writing setup is able to give rise to a localized photochromic effect, opening opportunities for 3D optical data storage.

In this perspective, Hu et al.^1^ recently published an interesting work in which they developed a new rare earth doped tungsten antimony phosphate glass composition. Under 473 nm laser irradiation, this glass exhibits a photochromic property appearing as a blue/dark aspect and related with a strong absorption band in the visible and near infrared range (500–1400 nm). Such photochromic effect could be tailored by both laser power density or laser irradiation time. The authors also demonstrated that 2D or 3D data storage can be achieved on this glass, being data writing ensured by laser irradiation and data reading accessible both by optical absorption or rare earth luminescence intensity measurements. The authors finally highlight that this new photo-modulated glass medium is promising for optoelectronic applications.

However, as authors of references 2 to 8, we would like to clarify and discuss important aspects of the paper by Hu et al.^1^.

As presented by the authors it seems that this glass composition was designed by the authors themselves. The main multicomponent nominal glass composition synthesized by Hu is 50WO_3_-39.5NaH_2_PO_4_-8BaF_2_-0.5Na_2_CO_3_-1Sb_2_O_3_-1EuF_3_ which corresponds to a final glass composition: 50WO_3_-39.5NaPO_3_-8BaF_2_-0.5Na_2_O-1Sb_2_O_3_-1EuF_3_. The authors do not justify the function of barium fluoride or addition of 0.5% Na_2_O. However, we already developed and described a photochromic glass of almost identical composition: 50WO_3_-40NaPO_3_-8.5BaF_2_-0.5Na_2_O-1Sb_2_O_3_^2^. The glass synthesis conditions used by Hu et al. are also the same we used in our work^2^ (1050 °C for 1 h in air). Besides, the authors introduced the key role of Sb_2_O_3_ addition on the final glass oxidation state and color but the use of this oxidizing agent and related redox mechanisms were also already described in our works^2,3^. In our case, the design of such a specific glass composition came from other previous works on tungsten fluorophosphate glasses^4,5^. These glass compositions were modified and optimized for photosensitive applications: the reason why we added 0.5% Na_2_O in our work refers to the fact that we used the nitrate precursor NaNO_3_ which reacts with Sb_2_O_3_ to form Sb_2_O_5_. This antimony (V) oxide decomposes during melting, releasing O_2_ in the melt and promoting oxidizing conditions^3^.

The photochromic effect in these tungsten antimony phosphate glasses under continuous visible laser irradiation was also studied in details and already reported by us^2,3^. The redox mechanisms, reversibility by thermal treatment, influence of the laser power or irradiation time and influence of the Sb_2_O_3_ content on the photochromism intensity probed by UV-Visible absorption as well as expected applications for 3D optical data storage presented by Hu et al. in their paper and supplementary materials were also already reported in our previous works^2,3,6–8^^.^

From our point of view, the main novelty of the paper presented by Hu et al. is the interesting use of Eu^3+^ emission and related intensity variations before and after irradiation as a way of data reading, even if this emission intensity variation is obviously due to the photochromic effect itself and stronger absorption of Eu^3+^ emission around 612 nm.

Therefore, it appears clear to us that nor the photochromic glass composition was developed by the authors nether the related photochromic effect was discovered by them. They rather used a known photochromic glass composition and used Eu^3+^ luminescence as an alternative way for optical data reading. Although reference 2 is cited in the introduction together with other works devoted to information storage in materials, it is not related with the studied glass composition and photochromic effect, giving the idea that both were developed from the research works by Hu et al.

Based on all these considerations and as developers of these photochromic materials^2^^,^^3^, we would like to express our disagreement with this research publication by Hu et al. without at least a proper recognition of the scientific original authorship.
